# Changing the obesogenic environment of severe mentally ill residential patients: ELIPS, a cluster randomised study design

**DOI:** 10.1186/s12888-014-0293-9

**Published:** 2014-11-25

**Authors:** Anne Looijmans, Frederike Jörg, Robert A Schoevers, Richard Bruggeman, Ronald P Stolk, Eva Corpeleijn

**Affiliations:** Department of Epidemiology, University of Groningen, University Medical Center Groningen, FA 40, PO box 30.001, 9700 RB Groningen, The Netherlands; Department of Psychiatry, University of Groningen, University Medical Center Groningen, Groningen, The Netherlands; Research Department, Friesland Mental Health Services, Leeuwarden, The Netherlands

**Keywords:** Severe mental illness, Residential patients, Cardiometabolic health, Obesogenic environment, Diet, Physical activity, Pragmatic trial

## Abstract

**Background:**

Severe mentally ill (SMI) patients have a reduced life expectancy of 13–30 years compared to the general population, largely due to an increased risk of cardiovascular mortality. Unhealthy lifestyle behaviours in SMI patients contribute to this increased risk. The obesogenic living environment of patients in residential facilities may even pose an extra risk. Although several studies have shown positive effects of lifestyle interventions on SMI patients’ weight status, studies including residential patients and their obesogenic environment are scarce. This paper describes the Effectiveness of Lifestyle Interventions in PSychiatry trial (ELIPS). The goal of this trial is to improve cardiometabolic health in severe mentally ill residential patients by addressing the obesogenic environment.

**Methods/design:**

The ELIPS study is a multi-site cluster randomised controlled trial (RCT) based on the principles of a pragmatic RCT. All residential and long-term clinical care teams of two large mental health care organisations in the North of the Netherlands serving SMI patients are invited to participate. The intervention is aimed at team level. Lifestyle coaches first develop a team specific lifestyle plan that tailors the ELIPS goals and protocol and then train teams on how to create a healthy environment and stimulate healthy behaviours in patients. After three months, teams take over the intervention after they have set out goals to achieve in the following nine months. In this phase, adherence to the lifestyle plan and pre-set goals is monitored. Patients in the control arm receive care as usual. Primary outcome measure is waist circumference at three and 12 months after baseline.

**Discussion:**

ELIPS is different from previously published lifestyle intervention studies in three ways. First, it follows the principles of a pragmatic design, which enables the examination of effects in everyday practice. Second, by implementing the intervention at team level, we expect lifestyle activities to be maintained when interventionists leave. Last, by targeting the obesogenic environment we create a prerequisite for any sustainable health improvement, as patients can only make healthy choices in a healthy living environment.

**Trial registration:**

Nederlands Trialregister NTR2720 (Dutch Trial Register, www.trialregister.nl). Registered 27 January 2011.

## Background

The life expectancy of patients with severe mental illness (SMI) is 13–30 years less than that of the general population [1]. This is largely due to an increased risk of cardiometabolic disease, including type 2 diabetes and cardiovascular disease. High rates of smoking, poor dietary habits, a sedentary lifestyle and side effects of type of antipsychotic medication are all modifiable risk factors that contribute to the increased mortality [2,3]. Numerous studies have investigated the effectiveness of lifestyle interventions in SMI patients [4-8]. These included single group interventions, non-randomized controlled trials and randomized controlled trials (RCTs). Although most studies are small and vary in their methodological quality, they all show that lifestyle interventions are feasible in SMI patients and can lead to weight loss and improvements in cardiometabolic health [9]. Mean weight losses across studies by treatment duration were 2.63 kg for 12- to 16-week interventions, 4.24 kg for 6-month interventions and 3.05 kg for 12- to 18-month interventions according to Gabriele et. al. [10].

In the current literature, lifestyle intervention studies in SMI residential patients are scarce. In their systematic review on exercise interventions for mentally ill inpatients, Stanton and Happell [11] found eight small studies with sample sizes varying from 12 to 39 patients. The somatic health of SMI patients living in residential facilities is not better than in SMI outpatients [11,12]. Moreover, residential patients’ living environment may pose an extra risk, as a lack of daily activities and an abundant provision of unhealthy food products may lead to an obesogenic environment. Swinburn et. al. [13] conceptualized the role of the obesogenic living environment as “the sum of influences that the surroundings, opportunities, or conditions of life have on promoting obesity in individuals or populations” (p. 564). Residential facilities seldom offer vocational rehabilitation activities, sports or physical activities, and residential patients are usually not involved in housekeeping chores. Patients may be offered meals from a central kitchen that are not always based on healthy nutrition guidelines, and snacks and other unhealthy food products are often readily available. It seems inefficient, and even unethical, to motivate patients to adopt a healthy lifestyle in an environment that stimulates unhealthy behaviour. While the obesogenic elements in residential patients’ living environment may play an important role in the development of weight gain, they yield possibilities for prevention and weight reduction as well. As far as we know, only one small intervention study (N = 37 at 12-month measure) aimed to tackle the obesogenic environment of patients [14].

Besides the focus on the obesogenic environment and the residential SMI population, our trial has another feature distinguishing it from other studies in SMI patients, and that is the so called ‘small change approach’. Large initial weight loss has been associated with greater weight gain later on [15]. Furthermore, even when weight loss is low (≤3%), lifestyle changes such as aerobic exercise can have beneficial effects on cardiometabolic risk factors such as insulin resistance, elevated blood lipids, blood pressure and glucose metabolism [16]. And last, smaller lifestyle changes are more likely to be sustainable, leading to increased health gain in the long run. In a small RCT, Sbrocco et. al. (1999) compared a small change approach in eating behaviour to a traditional behavioural weight loss program [17]. Initially, participants of the traditional program lost more weight than the small change group, but after nine months, the small change group continued to lose weight while the traditional behavioural therapy group started to regain weight. These results have been replicated recently in a US veterans study where small changes in both nutrition and physical activity led to weight loss and maintenance [18]. As far as we know, this small change approach has not been carried out in studies involving SMI patients, while they would particularly benefit from this approach considering the cognitive deficits and negative symptoms that are characteristic of this patient group.

Until now, most high quality lifestyle intervention studies in SMI patients have been RCTs. While a traditional measurement-blinded RCT is the preferred design to evaluate the efficacy of a lifestyle intervention, it also has important disadvantages. Often the most motivated patients are (unintentionally) selected for a trial in a laboratory setting. It has been shown that these patients are usually not comparable to patients in real-life settings [19]. Additionally, in a traditional RCT, lifestyle interventions are often strictly organised and supervised by interventionists, whereas in daily practice “health care interventions are seldom given under such circumstances”. (p. 2) [20]. The lack of external validity poses a threat to the effectiveness of interventions in daily practice, with not-so-motivated patients and without the dedicated attention of interventionists. For this reason, we designed a RCT with a pragmatic character to evaluate the effectiveness of a lifestyle intervention in a real-life setting with local staff members [20].

The aim of the current paper is to give a detailed portrayal of a clustered randomised controlled trial evaluating the effectiveness of a lifestyle intervention, the Effectiveness of Lifestyle Interventions in PSychiatry trial (ELIPS). This combined diet and physical activity lifestyle intervention is designed to improve the obesogenic environment of SMI residential patients. It is hypothesised that changing the obesogenic environment by engaging patients in physical activities and offering them the choice for healthy products, will improve cardiometabolic health in this patient group.

## Methods

ELIPS is a multi-site trial designed according to the principles of a pragmatic RCT to allow evaluation of the intervention in real-life care facilities. Details are described below according to the CONSORT statement for cluster randomised trials [21] and the extension of the CONSORT statement for pragmatic trials [22]. The Medical Ethical Committee for Research in Mental Health Care (Metigg) approved the study protocol. The trial is registered in the Dutch Trial Registry (Nederlands Trialregister NTR2720, www.trialregister.nl. Registered 27 January 2011).

### Participants

All residential and long-term clinical care teams of two large mental health organisations in the North of the Netherlands, a catchment area of 1.2 million inhabitants, are invited to participate in the study (N = 30). One residential team exclusively serving patients suffering from Korsakov’s syndrome is excluded. Team leaders are informed by an invitation letter followed by a meeting to explain the study. Teams are visited by the project leader to explain the aims of the study and answer questions. Teams serve severe mentally ill patients, often with a diagnosis of schizophrenia or other psychotic disorder, most of whom have been living in residential facilities for at least 10 years. Caseload sizes vary from 20 to 65 patients per team. In the Netherlands, patients living in residential facilities live as independently as possible, with a team of professionals nearby. Patients are housed alone or in small groups in a community neighbourhood. Long-term clinical care facilities provide more intensive professional care and assistance in activities of daily living. Housing is provided in the sheltered and safe environment of the mental health care organisation.

All patients are invited to participate, as they are all at increased risk of unhealthy lifestyle habits due to their obesogenic living environment, and all patients are considered to have an increased cardiometabolic risk. Exclusion criteria are age below 18, pregnancy and being unable to perform physical activity tests (e.g. wheelchair user). Patients not taking part in the yearly routine outcome monitoring (ROM, see ‘outcomes’), that is used for this study to monitor intervention effects, will not be included in the analysis. In the mental health organisation Lentis (~50% recruitment area) only patients using antipsychotic medication are routinely monitored. Therefore, patients from Lentis not using antipsychotic medication are excluded.

### Intervention

The ELIPS intervention is aimed at team level and focuses on achieving established ELIPS lifestyle goals. These goals are: 1. At least two physical activities per week, of which at least one moderately intensive; 2. At least three changes in daily food supply that favour health; 3. A weekly food workshop, in which patients learn to buy, cook and eat healthy food products; 4. A sustainable change in the obesogenic environment on organisation level. The ELIPS goals and subgoals are presented in Table 1. The intervention is implemented by lifestyle coaches; each team has two lifestyle coaches at its disposal. Before the start, lifestyle coaches receive two days of intensive training which consists of lectures and interactive sessions on SMI and its symptoms (positive and negative psychotic symptoms, depression), metabolism, healthy food products, physical activities suitable for SMI patients, motivational interviewing, the development over time of the obesogenic environment and ways to tackle it.

**Table 1:** **Lifestyle goals of ELIPS intervention and examples from practice**

**Goal**	**Subgoal and example**
**1. Minimally two physical activities per week, one of them being intensive**	
	1.1 increase in daily physical activity on individual level
	*E.g. counselling conversations during a walk outside rather than sitting in the office; patients walk/cycle to shop for own groceries; patients walk/cycle to work rather than taking the bus.*
	1.2 increase in daily physical activity on group level (low intensity)
	*E.g. organise group walks one or twice a week.*
	1.3 participation in more intensive physical group activity (organised, medium intensity)
	*E.g. organise a weekly football activity, organise a WII-sports activity, visit a fitness centre once a week, go for a swim once per two weeks.*
**2. Minimally three improvements in food supply**	
	2.1 decrease in intake of saturated fat and trans fatty acids
	*E.g. replace high-fat cheese by low-fat cheese; limit consumption of sweetened dairy products; limit cake consumption (luxury cookies only on Sundays).*
	2.2 increase in intake of fibres
	*E.g. replace white bread by whole wheat bread; replace white rice/pasta by whole rice/pasta; serve fruit and vegetables during activities.*
	2.3 decrease in energy intake
	*E.g. replace products by light versions; replace butter by (diet) margarine; replace almond paste cake by gingerbread.*
	2.4 decrease in intake of salt
	*E.g. buy fresh vegetables instead of canned vegetables; replace crisps by vegetables (cucumber); offer snacks in small portions, and/or only in the weekends.*
**3. Minimal one food focused activity per week**	
	*E.g. create a daily fruit moment; make a shopping list together; buy healthy groceries together; cook a healthy meal together; play a food quiz; make smoothies; learn to decipher the list of ingredients.*
**4. Adjustment of the obesogenic environment on organisational level**	
	*E.g. limit access to food cupboards, adjust food supply in canteen (selling fried snacks only twice per week); provide a gym; set up contracts with fitness centres; purchase a WII sports; prepare breakfast every morning; put nutrition and physical activity standard on the team meeting’s agenda.*

The trial consists of a preparation phase, an implementation phase and a monitoring phase. An overview of phase-specific tasks is given below. The team specific interventions are outlined in the one-month preparation phase. During this period, lifestyle coaches become acquainted with team members, patients and the location. The coaches screen which healthy and unhealthy behaviours and lifestyle activities take place and seek possibilities for improvement that comply to the ELIPS goals. To increase activity participation and sustainability, lifestyle coaches make a list of activities that patients and team members find appealing. Structured protocols describe planning of lifestyle activities (i.e. minimal two physical activities and one food workshop per week) and form the basis of each intervention to insure that team specific interventions are compatible and all teams pursue the ELIPS lifestyle goals. The team specific lifestyle plan includes a detailed program of exercise and food activities according to the ELIPS goals and is adjusted to the team’s opportunities, patients’ preferences and the logistic possibilities of the location. This tailor-made lifestyle plan colours this pragmatic cluster randomised trial.

Description of tasks and schedule of ELIPS intervention:

*-1 - 0 months Preparation phase*
  - Lifestyle coaches visit intervention teams to become acquainted with the team, patients and location.
  - Lifestyle coaches explore which (un)healthy behaviours and activities take place.
  - Lifestyle coaches inquire which lifestyle activities patients and teams prefer and examine logistic possibilities for these activities.
  - Lifestyle coaches create a team-specific lifestyle plan based on ELIPS lifestyle goals, structured protocols and the preferences and possibilities of the team and patients.
  - Baseline measures: ROM-nurses carry out ROM measurements, research assistants carry out additional measures. See Table 2 for an overview of measurements.
- *0 - 3 months Implementation phase*
  - Lifestyle coaches organise lifestyle activities and workshops. The team is involved in the organisation and motivates patients to participate. Lifestyle coaches first demonstrate the activities, then co-organise them out together with the team, while towards the end, they supervise team members who, over time, are made responsible for carrying out the lifestyle activities themselves.
  - Lifestyle coaches transfer their knowledge and skills of healthy behaviours to the team.
  - Lifestyle coaches assist the team in setting concrete goals in order to improve or maintain a healthier lifestyle after the lifestyle coaches have gone.
  - Structural changes to adjust the obesogenic environment are explored and implemented.
- *3 - 12 months Monitoring phase*
  - 3-months measures: ROM-nurses carry out ROM measurements, research assistants carry out additional measures. See Table 2 for an overview of measurements.
  - Team continues lifestyle activities, aiming to achieve the pre-defined goals.
  - Start monthly monitoring visits. A lifestyle coach evaluates with the team the pre-defined goals, detects obstacles in achieving these goals, solves observed obstacles. If necessary, recommendations for improvements are made.
  - 12-months measures: ROM-nurses carry out ROM measurements, research assistants carry out additional measures. See Table 2 for an overview of measurements.
  - 12-months monitoring visit: final monitoring wherein goals are evaluated and recommendations are made to sustain the lifestyle activities.

In the three months intervention phase the team specific intervention is implemented. Lifestyle coaches first demonstrate the activities, then carry out the activities together with team members and finally supervise while team members carry out the activities themselves. During the entire implementation phase, lifestyle coaches train teams how to create a healthy environment for their patients and stimulate a healthier lifestyle in their patients. Lifestyle coaches transfer their knowledge and skills to the team members by demonstrating exercise and food activities as well as using motivation and counselling techniques to stimulate patients to adopt a healthier lifestyle. Exercise activities are for instance walking, low-intensity sports such as jeu de boules, badminton, table tennis, bicycling. The intensity and frequency of the exercises will gradually increase. Food workshops could be for example cooking a vegetable soup together with patients, making smoothies, teaching how to read a list of ingredients, demonstrating the amount of sugar in soft drinks and juices and doing affordable fruit and vegetable shopping in the market place together. Examples of adjusting the environment are restricting access to food cupboards, setting up contracts with local gyms, placing fruit on the table. More practical examples are presented in italics in Table 1. At the end of the three months implementation period, the team sets goals to achieve in the following 9 months. During this 9 month monitoring phase a lifestyle coach visits all intervention teams monthly and will discuss with the team and team leader whether goals are achieved, if there are any barriers in achieving the goals and look for ways to remove the obstacles.

The intervention has two distinct features that are not described in lifestyle interventions presented in the literature [9]. First, the intervention is implemented at team level. We hypothesise that team members will be able to induce the largest change in their patients’ lifestyle behaviours as they already have a relationship with patients. Moreover, it will be easier to continue healthy lifestyle activities after the intervention period, when these activities are imbedded in the team and in the team’s schedule. Also, team members might become stimulated to adjust their own lifestyle as well and in doing so, set a good example. The second distinct feature of the intervention is the focus on changing the obesogenic environment. Stimulating patients to engage in healthy lifestyle behaviour can only be a success when the environment offers sufficient possibilities for healthy lifestyle choices. Sport facilities and exercise activities must be available, as well as healthy food products. Likewise, the environment can discourage unhealthy behaviours, for instance by narrowing the opening hours of canteens serving fried snacks. It is hypothesised that an intervention with these distinct features will lead to changes in lifestyle behaviour in patients that are highly sustainable.

### Control group

The patients in the control arm receive care as usual. No ELIPS lifestyle intervention activities will be carried out in the control teams. Teams or individual team members are not prohibited to discuss healthy lifestyle behaviours with their patients or organise healthy lifestyle activities. All healthy lifestyle activities will be documented and new initiatives are reported to the researchers. When ethically and logistically possible, researchers will ask teams to delay implementation of these activities until the end of the study. Monitoring visits by lifestyle coaches will establish possible changes in intensity or frequency of existing lifestyle activities during the study.

### Objectives

The primary objective is to determine the effect of the ELIPS intervention on body fatness defined by waist circumference at 3 and 12 months. We hypothesise that the ELIPS intervention group will have a reduced or stabilized waist circumference whereas the waist circumference of the control group will increase. The secondary objective is to determine the effect of the ELIPS intervention on other cardiometabolic risk factors, i.e. blood pressure, lipids and glucose metabolism. Furthermore, the aim is to determine the effect of the ELIPS intervention on lifestyle behaviours (physical fitness, physical activity, dietary habits), mental health (positive and negative psychotic symptoms, depressive symptoms), quality of life and psycho-social functioning of patients. For all these objectives, the difference in effect across age groups and gender will be studied. In view of the pragmatic trial, a process evaluation on the degree and quality of the implementation of the intervention and changes at team and organisation level is foreseen. The outcomes of this evaluation will be used to investigate differences in effectiveness between teams. Lastly, the effect of the ELIPS intervention is investigated in terms of cost-effectiveness. The cost-effectiveness of lifestyle interventions has not been researched in severe mentally ill residential patients and this study will give an indication of the costs related to health improvements in this population.

### Outcomes

Measurements are performed on patient and team level. A detailed description of the measures is given below and an overview of all measurements is given in Table 2. The measurements will be performed at baseline and three and twelve months after the start of the intervention. A large part of the measurements are performed routinely as part of standard care. These Routine Outcome Monitoring (ROM) screenings are implemented in all mental health care organisations in the North of the Netherlands. Trained nurses visit patients yearly after individual appointments have been made or schedules are set up by team coordinators. Results are recorded in the patient record form and discussed with the patient. When ROM screenings were first implemented, patients were asked for informed consent to use their ROM data for research purposes. Only anonimized data are used for scientific research, and the procedure was approved by the Medical Ethical Committee of the University Medical Center Groningen. For the ELIPS data collection, two regular, yearly ROM screenings will be used (baseline and 12 months follow up) and an additional ROM screening will be carried out at three months for which patients receive a small participation fee (€5,00).

**Table 2:** **ELIPS Trial measurements overview**

		**Baseline**	**3 months**	**Monthly, during monitoring phase**	**12 months**
**Measurements on patient level**					
General information	Age, gender, birth year, diagnoses, year of first psychosis	X			
Physical measures	Height	X	X		X
	Weight	X	X		X
	Waist circumference	X	X		X
	Blood pressure (systolic, diastolic, pulse)	X	X		X
	Body fat (%)*	X	X		X
Lab test	Lipids (Total cholesterol, LDL-cholesterol, HDL-cholesterol, triglycerides)	X	X		X
	Glucose metabolism (glucose, HbA1c)	X	X		X
	Clinical parameters (CRP, ASAT, ALAT, urea, creatinin, TSH, leukocytes)	X	X		X
Psychological measures	CDSS	X	X		X
	PANSS	X	X		X
	HoNOS	X	X		X
	MANSA	X	X		X
Lifestyle habits	Functional physical fitness	X	X		X
	(six-minute walk test)*				
	Daily physical activity (SQUASH)*	X	X		X
	Food frequency questionnaire (adapted to patient population)				
Cost-effectiveness	Dutch care consumption questionnaire*	X	X		X
	SF6D*	X	X		X
**Measurements on team level**					
General information	Age, gender, birth year, level of education, number of years working in psychiatry, function*	X	X		X
Process evaluation	Semi-structured interview team leader*	X	X	X**	X
	Semi-structured interview staff*	X	X	X**	X
	Semi-structured interview patients*	X	X	X**	X
	Observatory list*	X	X	X**	X
Staff questionnaire	Knowledge on diet and physical activity, attitude towards lifestyle changes in patients, self-efficacy in addressing lifestyle issues with patients*	X	X		X

*Measurement is not part of ROM screening. Additional measures are collected by research assistants.

**Measures are collected at intervention teams only.

Some measurements are not part of ROM and will be carried out by research assistants, mostly the lifestyle coaches. Research assistants will receive one day training to study protocols and procedures and to practice assessing the functional physical fitness in patients with the six-minute walk test, assessing the body fat percentage with the Bioelectrical impedance analysis, measuring height and weight, assessing food habits with a food questionnaire based on Dutch guidelines for a healthy diet which is adapted to this population, and questionnaires and interviews with patients and staff for process evaluation. All measurements will be taken on site or in a location that is familiar to the patient (e.g. patient’s own room, nurses’ office). Research assistants contact patients directly to set up an appointment or staff members act as intermediaries to facilitate setting up appointments. Appointments for interviews with staff members are scheduled by research assistants and staff members.

### Measurements on patient level

Patient measurements include cardiometabolic health, disease history, medication use, mental health, psychosocial functioning, quality of life, lifestyle habits, care consumption and general information (i.e. age, gender).

Cardiometabolic health. The physical measurements include waist circumference, height, weight, pulse and systolic and diastolic blood pressure. Patients visit a (hospital) laboratory that collects a blood sample, if possible in fasting state for levels of lipids (total cholesterol, LDL-cholesterol, HDL-cholesterol and triglycerides), glucose metabolism (glucose, HbA1c) and clinical parameters (i.e. CRP, ALAT, ASAT, urea, creatinine, TSH, leukocytes) and vitamin D status. Measurements are taken following standard ROM protocols. Waist circumference is measured in duplicate using a flexible nonstretching tape halfway between the iliac crest and lowest rib in standing position at the end of an expiration. Weight is measured by calibrated scales (Seca, model 813) in light clothing without shoes or jackets. Measurements for height will be available from multiple measurements of both ROM nurses and research assistants. The highest height will be used unless patients wear shoes, then the highest height without shoes is used. Pulse and systolic and diastolic blood pressure are measured after 5 minutes’ rest in sitting position, using a blood pressure monitor (BOSO medicus control). Body fat percentage is measured in standing position [23] by bioelectrical impedance analysis (BIA) in triplicate using a single-frequency bioimpedance analyzer (Model BIA 101, AKERN Srl, Italy) [24,25].

Mental health. During an interview, trained nurses administer the PANSS (Positive and Negative Syndrome Scale, [26]) and the CDSS (Calgarary Depression Scale for Schizophrenia, [27]). Prior to the interview, patients fill in the MANSA, a self-report questionnaire about patients’ Quality of Life [28] and uncertainties can be discussed during the interview. The HoNOS (Health of the Nations Outcome Scale, [29]) is an observation scale of psycho-social functioning and is scored by the case manager or team.

Lifestyle habits. Functional physical fitness is assessed by research assistants with a detailed protocol for the six-minute walk test [30], which contains standard phrases and time points for encouragement and has been validated in patients with schizophrenia [31]. The intraclass correlation coefficient was 0.96. Although a mild practice effect was detected for a three-day interval, this is unlikely to persist for a three–month interval. The test is conducted indoors. Patient staff is allowed to walk along with patients if necessary. Daily physical activity is assessed using the Dutch validated SQUASH questionnaire [32]. Nutritional habits are estimated using a semi-quantitative food frequency questionnaire with items based on a screening questionnaire for healthy eating habits of the Netherlands Nutrition Center according to the Dutch guidelines for a healthy diet [33] and adapted to this population. The questionnaire will be used to assess changes in dietary habits on food group level. It is not specifically validated in SMI patients and can and will not be used to derive quantitative estimates of total energy, macro- or micronutrient intake.

Cost-effectiveness parameters. Care consumption is estimated with the Dutch care consumption questionnaire [34], which is adapted to the context of the current study. Quality adjusted life years (QALYs) will be the primary outcome measure in the cost-effectiveness analysis. In order to estimate QALYs, utility scores will be derived from the SF12, using the SF6D algorithm [35,36].

### Measurements on team level

Measurements at team level include lifestyle knowledge and attitudes from staff, level of support of team managers, and process evaluation. Mental health care professionals are asked to fill in questionnaires for process evaluation and staff characteristics as potential determinants of successful intervention at baseline, and three and 12 months after start of the intervention.

Process evaluation. One research assistant visits all teams (both control and intervention) and will interview the team leader, staff and patients separately, using semi-structured interviews, at baseline, three months and 12 months to determine which lifestyle activities are initiated and sustained. These measurements are in addition to the monthly monitoring visits that have a more advisory character. The interviews contain questions on organisational aspects (e.g. is anyone in the team specifically responsible for the lifestyle activities and if yes state name) as well as on lifestyle-related activities. These activities will be reported in great detail (what is being organised, for how long, how often, how many participants from the housing facility participate, and more). Questions, translated from Dutch, are for example “How many times per week are physically intensive activities offered?”, “What have you changed in food supply?”, “What type of food workshops have you organised?” and “Are patients stimulated to do household chores?”. During these visits, the research assistant will screen kitchen cabinets, refrigerator and shared living rooms to determine the level of ‘obesogenic environment’ (e.g. fruit on the table, healthy products in store or products with reduced fat and energy in the cabinets and refrigerator, number of exercise activities written in the team’s calendar). The information is combined to establish adherence to the lifestyle plan and the degree and quality of implementation of the intervention.

Mental health professionals’ characteristics. Data on age, education and experience are collected at baseline. At baseline, and three and 12 months after the start of the intervention, at least three members of the team will be asked to fill in questionnaires to rate their knowledge on diet and physical activity, attitude towards lifestyle changes in patients, and self-efficacy in addressing lifestyle issues with patients (Staff questionnaire). The questionnaire is developed based on existing questionnaires from the Netherlands Nutrition Center to evaluate lifestyle knowledge. Questions on attitude and self-efficacy are based on the ACE-model, better known as the ASE-model. This model describes the relationship between a persons’ attitudes, social influences and self-efficacy, and his or her behaviour. Questions are adapted to fit the study design and patient group [37,38].

### Sample size

The primary objective in this study is to detect a difference in waist circumference with clinical significance. Sample size calculations show that to detect a significant change of −5% waist circumference [39] (alpha =0.05, power 0.90), 219 patients are needed in both intervention and control arm. Research in psychiatric patients usually shows a high drop-out rate. However, since in this project patients are living intramurally and most measurements are part of standard care, it is expected that the drop-out rate, once having signed an informed consent, will not exceed 10%. Therefore the target number of patients to include in both intervention and control arm is 240 (N = 219 + 10% drop-out). In both mental health organisations together, approximately 1000 eligible SMI patients live in residential or long-term clinical care facilities. Results from a pilot study show that two thirds of patients will successfully participate in the measurements which means that the two organisations will suffice to include the necessary 240 patients per arm. This number will also be sufficient to demonstrate for example a 4.7% reduction in plasma glucose. The clustered nature of the trial requires some extra thoughts on loss of power in multilevel analyses. However, as we a have a large number of clusters (13 clusters; see below) and a large number of patients per cluster (range from 44 to 130), the interdependency of observations will be limited. This may justify using individual patient rather than clustered sample size calculations [40].

### Randomisation

After recruitment, teams are randomised to intervention or control condition. To ensure comparability of teams in the intervention and control arm, clusters of comparable teams are made based on health care organisation, level of intensity of care (long-term residential or long-term clinical care), caseload size and location (urban or rural). Each cluster consists of two comparable teams in terms of these variables; one cluster contains five comparable teams. Of each cluster, the teams are randomly allocated to one of the two conditions by a computerised random number generator. A research nurse not involved in the study reveals the results of the allocation. All participating teams are randomised simultaneously making allocation sequence concealment unnecessary.

Blinding of teams and patients to the allocation condition is not possible due to the nature of the intervention. Research nurses assessing the outcomes in the ROM screenings are blinded to the intervention allocation. The research assistants taking the six-minute walk test, body fat test and lifestyle questionnaires in patients and staff are not blinded to the intervention at the baseline and 3 months measurements. Outcome measures at 12 months after baseline are however carried out by different research assistants that are not informed of the allocation status of the teams and patients. All post-intervention measures at 12 months after start of the intervention will thus be obtained blinded.

### Statistical methods

Data will be analysed with intent-to-treat analysis because all patients within intervention teams are expected to be exposed in some extent to the adjusted obesogenic environment. A multi-level linear mixed model will be used in order to evaluate the effect of intervention group on waist circumference at 3 and 12 months after baseline, with intervention group (i.e. belonging to an intervention or control team) being the main independent variable. The model accounts for the clustering of teams and the longitudinal character of the trial. Adjustments for baseline scores will be made and interactions with age and gender will be studied. Secondary outcomes, other cardiometabolic risk factors, lifestyle behaviours, mental health, quality of life and psycho-social functioning, will be analysed in the same way as waist circumference.

The number of achieved ELIPS goals, the degree of additional changes in the environment, the stability of teams, and knowledge, attitudes and self-efficacy of teams and team leaders towards a healthy lifestyle will be scored to give insight in the variation and quality of implementation of the intervention per team. Subgroup analyses will be done to find out if there are differences in outcomes between patients within teams with more of less successful implementation of the intervention. This might also give an idea of minimal conditions needed for this intervention to be implemented to a certain degree.

An economic evaluation will be conducted to assess the cost-effectiveness of the lifestyle intervention compared to care as usual in severe mentally ill residential patients. The analysis will be performed from a societal perspective; costs in and outside the healthcare sector will be included. Results will be expressed in terms of incremental costs per percentage change in waist circumference. Furthermore, a cost-utility analysis will be conducted with the QALY (Quality Adjusted Life Years) as primary outcome measure. Finally, potential long-term economic consequences due to reduced incidence of diabetes and other cardiovascular diseases will be explored.

Missing data and drop-outs will be analysed and accounted for by multiple imputation if the assumption of data missing at random (MAR) is not violated.

## Discussion

Several diet and physical activity interventions have successfully improved cardiometabolic health in SMI patients [9]. However, large randomised controlled trials studying lifestyle interventions in residential patients are lacking, even though these patients might be more vulnerable for unhealthy lifestyle behaviours due to an obesogenic living environment. To our knowledge, only one study addressed the environment of patients with schizophrenia or schizoaffective disorder hospitalised for one year or longer [14]. Staff members were involved in this study and results show no significant differences in BMI between intervention and control patients after 12 months. However, the sample size of this study was small with 37 patients included in the 12 months measures.

The distinctive features of the ELIPS trial compared to other lifestyle interventions in SMI patients are the focus on modifying the obesogenic environment of residential patients and the implementation of the intervention on team level. Changing the obesogenic environment is expected to have long-term effects on patients’ health due to the sustainable character of these changes. Interventions that include modification of the environment to stimulate healthy behaviour show positive results: there is potential for improving dietary habits on the work site [41] and, combined with education, it leads to improvements in dietary habits and waist circumference in men with substance addictions in residential settings [42]. Implementing the intervention on team level is expected to lead to maintenance of health behaviours after the intervention stops. In the study of Álvarez-Jiménez and colleagues [43] a three month behavioural intervention led to less weight gain in first-episode psychosis patients compared to controls, but this effect had diminished three months after the intervention ended. The ACHIEVE trial [8] showed significant weight reduction 18 months after the start of a lifestyle intervention in overweight and obese SMI patients. However, during this 18 month period several aspects of the intervention, such as individual visits and physical activity classes, were led by the interventionists. This increases the costs of the intervention and it remains to be seen whether the weight loss is maintained when the sessions led by interventionists stop. Most experts in the obesity field argue that the key to successful stabilisation of new behaviours is long-term treatment [44]. ELIPS will implement the intervention on team level, by coaching team members in creating a healthy living environment for their patients and in stimulating them to adopt healthy lifestyles. As the intervention is applied in daily routine and fits in the team’s schedule, it is expected that the effect of the intervention maintains when lifestyle coaches leave.

A strength of the inclusion on team level is that patient selection bias is decreased. Patients are automatically invited to participate, except when they meet the exclusion criteria (age <18, pregnancy or being unable to perform physical activity tests). The target sample is large with in total approximately 1000 eligible patients. The multi-site character of the trial with all 29 eligible teams in the North of the Netherlands being invited, the setting of teams in both urban and rural areas, and the number of eligible patients per team will lead to the inclusion of most of the SMI residential patients in the North of the Netherlands. This representative sample, together with the implementation of the intervention in real-life settings, emphasises the generalisability of the results of the ELIPS trial.

Advantages of using measures conducted as part of standard care are the reduced costs and the limited impact on patients. Measurements taken during the ROM screening (PANSS, CDSS, HoNOS and MANSA) are widely validated. Currently, feasible and validated methods to assess dietary intake in SMI patients, however, are lacking. It will be quite a challenge to adapt and validate a widely applicable method to reliably assess dietary intake in SMI patients, considering their cognitive deficits.

When randomisation is successful, both conditions will consist of teams that are and teams that are not already interested in or acquainted with healthy lifestyle matters. Although patients in the control teams receive care as usual, some of these teams might be or become active in offering lifestyle activities and in stimulating healthy behaviours in their patients, even at baseline [45]. Therefore, in both conditions, all lifestyle activities are evaluated at baseline, and at three and twelve months follow up. In the control condition, teams are kindly requested to discuss new or planned initiatives with the researchers to see if delay of initiatives is possible. Baseline patient characteristics are checked to ensure comparability of patients in control and intervention teams. If necessary, corrections for baseline differences will be made during analysis.

ELIPS is a lifestyle intervention aiming to improve cardiometabolic health in severe mentally ill residential patients by adjusting the obesogenic environment. If the ELIPS intervention proves to be successful, the results will have large external validity as it is performed in a real-life setting with a representative sample. By tackling the obesogenic environment of long-term residential patients, we expect to achieve an improved health status that can be maintained.

## Abbreviations

SMI: Severe mentally ill
BMI: Body mass index
RCT: Randomised controlled trial
